# Supplementary material for: Torsion is a dynamic regulator of DNA replication stalling and reactivation
Source: Nat Commun. 2025 Nov 26;16:10543. doi: 10.1038/s41467-025-65567-5 (PMC12658067; doi:10.1038/s41467-025-65567-5)
Supplement: Supplementary file 1 — Supplementary Information [file 41467_2025_65567_MOESM1_ESM.pdf]

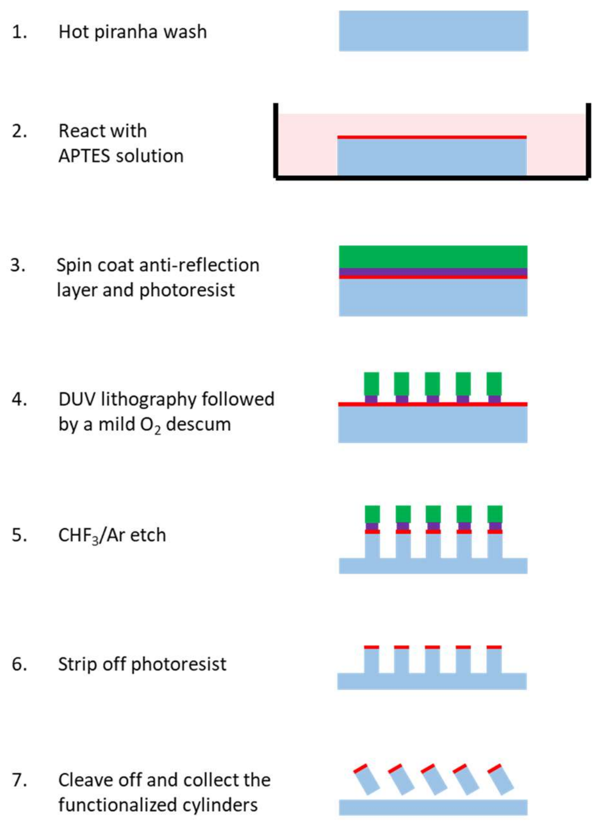

Supplementary Fig. 1. Quartz cylinder fabrication.

This protocol is revised from our previous methods[1,2]. 1 and 2, the x-cut single-crystal quartz wafer is cleaned with a hot piranha wash and derivatized by reaction with 3-aminopropyltriethoxysilane (APTES) solution (red). 3, the wafer is spin-coated with an anti-reflection layer (purple) and photoresist (green). 4, the photoresist is exposed with deep UV (DUV) photolithography followed by a mild oxygen (O_2) plasma descumming treatment. 5, the pillars are etched into the quartz wafer with a CHF_3/Ar plasma etch. 6, the photoresist is stripped from the cylinders, exposing the derivatized top surface that will later be used for specific functionalization. 7, the cylinders are mechanically separated from the wafer by scraping with a razor blade.

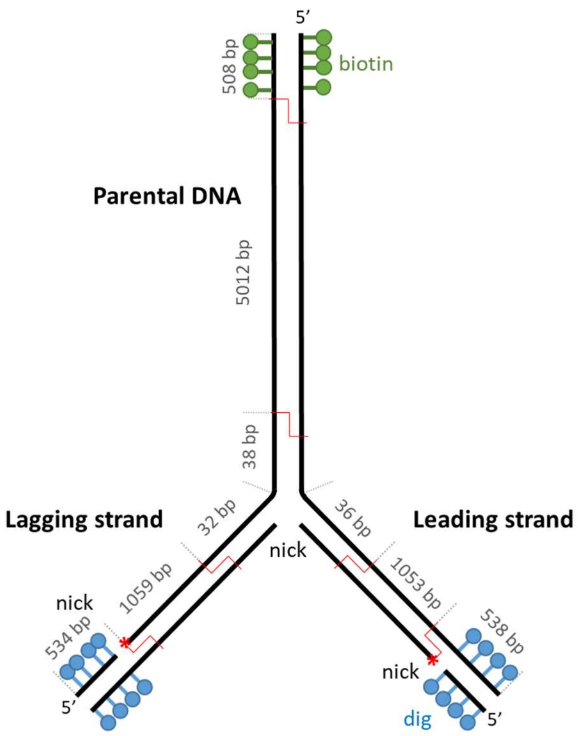

Supplementary Fig. 2. The Y-shaped DNA substrate for replication.

This substrate consists of three segments of DNA brought together at a three-way junction that resembles a replication fork. The parental DNA contains a multi-labeled biotin adapter at the end to enable torsional constraint to a surface of interest. The leading and lagging strands each contain a multi-labeled digoxigenin adapter at the end. Each strand can freely rotate around its own helical axis due to the presence of a nick near the adapter. This nick was introduced to resemble the torsional state *in vivo* since prior studies suggest that each strand can rotate around its own helical axis[3].

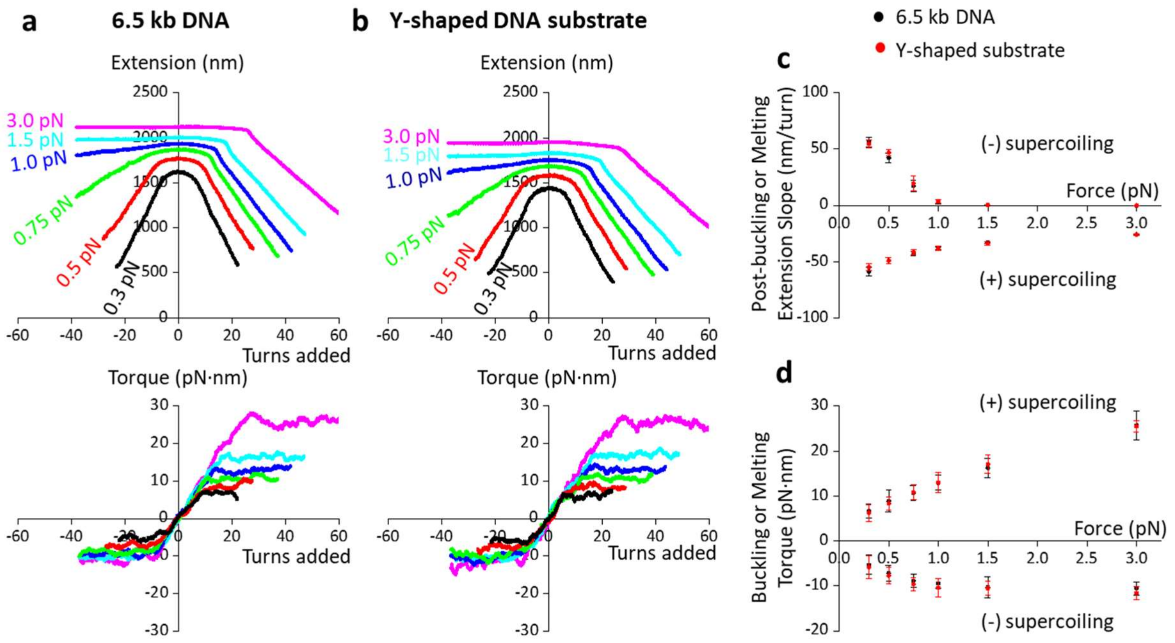

Supplementary Fig. 3. Torsional mechanics of the Y-shaped DNA substrate.

We compared the torsional mechanics of the Y-shaped DNA substrate (Supplementary Fig. 2) and a linear DNA of similar length (6.5 kb DNA) that we previously employed[4]. As shown below, we found that the two DNA substrates show nearly identical buckling properties, suggesting that the presence of the leading and lagging anchoring strands do not significantly alter these properties.

a. Extension and torque versus turns for the 6.5 kb DNA. Data shown are collected from *N* = 16 individual DNA substrates.

b. Extension and torque versus turns for the Y-shaped DNA substrate. Data shown are collected from *N* = 15 individual DNA substrates.

c. Post-buckling or melting extension slope versus force. At each force, the slope is obtained from a linear fit to the post-buckling or melting region of the extension for each trace with the error bar being SD of the fit parameter.

d. Post-buckling or melting torque versus force. At each force, the torque is obtained from a horizontal line fit to the post-buckling or melting region of the torque for each trace with the error bar being the SD of the fit parameter.

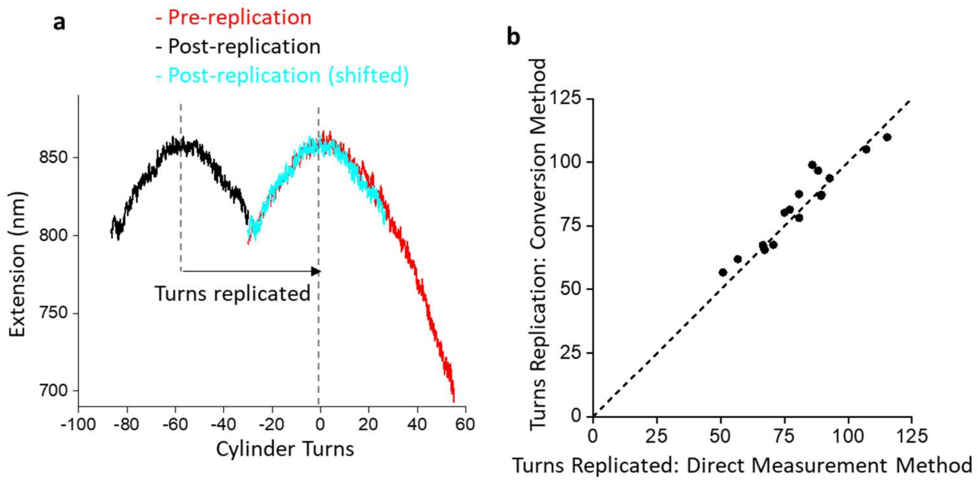

Supplementary Fig. 4. Replisome fork position determination.

During a stalling experiment (Fig. 2), we measured the DNA extension, which is then used to determine the real-time fork position (Methods). This conversion method relies on a calibration curve, which is established before the start of each replication measurement. This method assumes that changes in DNA extension are primarily a result of DNA supercoiling introduced by replication progression. To evaluate the accuracy of this conversion method, we performed a direct measurement of total turns replicated at the end of the replication.

a. An example trace illustrating the method for direct determination of the total replication turns at the end of a measurement. In this trace, before replication start, we measured the extension versus turns relation of the Y-shaped DNA substrate under a constant trap height (red). The peak position of this curve represents the equilibrium position of the substrate and was initially centered at zero turns. After replication, we re-measured this relation (black), which exhibits a shift of the equilibrium position to the left because replication added extra turns to the substrate. The shift in the equilibrium position provides an accurate measure of the number of replicated turns, as the shape of the curve near the equilibrium position remains minimally unchanged after replication (cyan). The conversion to the number of base pairs replicated is straightforward (10.5 bp/turn). Note that this method can only be used for traces that show minimal replication activity during the post-replication measurement, as replication activity distorts this relation.

b. Comparison of the turns replicated at the end of a stalling measurement using the conversion method and the direct measurement method. Each data point is from a unique trace. Data points falling along the dashed grey guideline (slope = 1) should indicate that the two methods are in close agreement. This plot suggests that the fork position conversion method is accurate within 10%, suggesting the conversion method is a relatively accurate method for real-time fork position tracking.

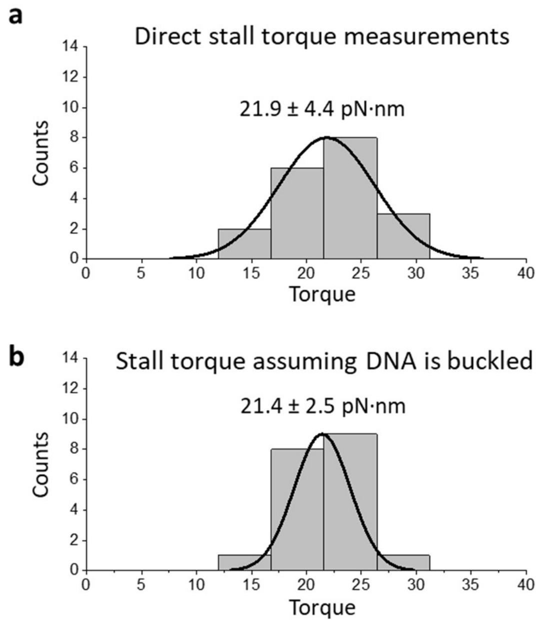

Supplementary Fig. 5. Replisome stall torque measurements.

In Fig. 2, we directly measured the stall torque of the replisome using the torque detector of the AOT. This method is necessary especially when the parental DNA is not buckled. If the parental DNA is buckled, the stall torque can be either directly measured or obtained from the measured force using the buckling torque versus force relation (Supplementary Fig. 3d), because a buckled DNA has a well-established torque-force relation[5,6,1]. We have previously used the latter method to determine the torque that RNA polymerase generates during stalling under torsion[7,8], since the noise in force measurements is significantly less than the noise in the torque measurements. Below, we provide evidence that these two methods give similar stall torque values for a replisome, indicating that the parental DNA is buckled when the replisome is stalled. For torque data shown in Fig. 3a and Fig. 4b-d, the latter method is used.

a. Stall torque measured directly by the torque detector of the AOT. This histogram is identical to the wt replisome panel shown in Fig. 2. The mean stall torque is 21.9 ± 4.4 pN·nm (mean ± SD) from N = 19 traces.

b. Stall torque assuming the parental DNA is buckled. For the same set of traces used in **a**, the stall torque is obtained from the measured force at the stall, converted using the buckling torque versus force relation (Supplementary Fig. 3). The mean stall torque is 21.4 ± 2.5 pN·nm (mean ± SD).

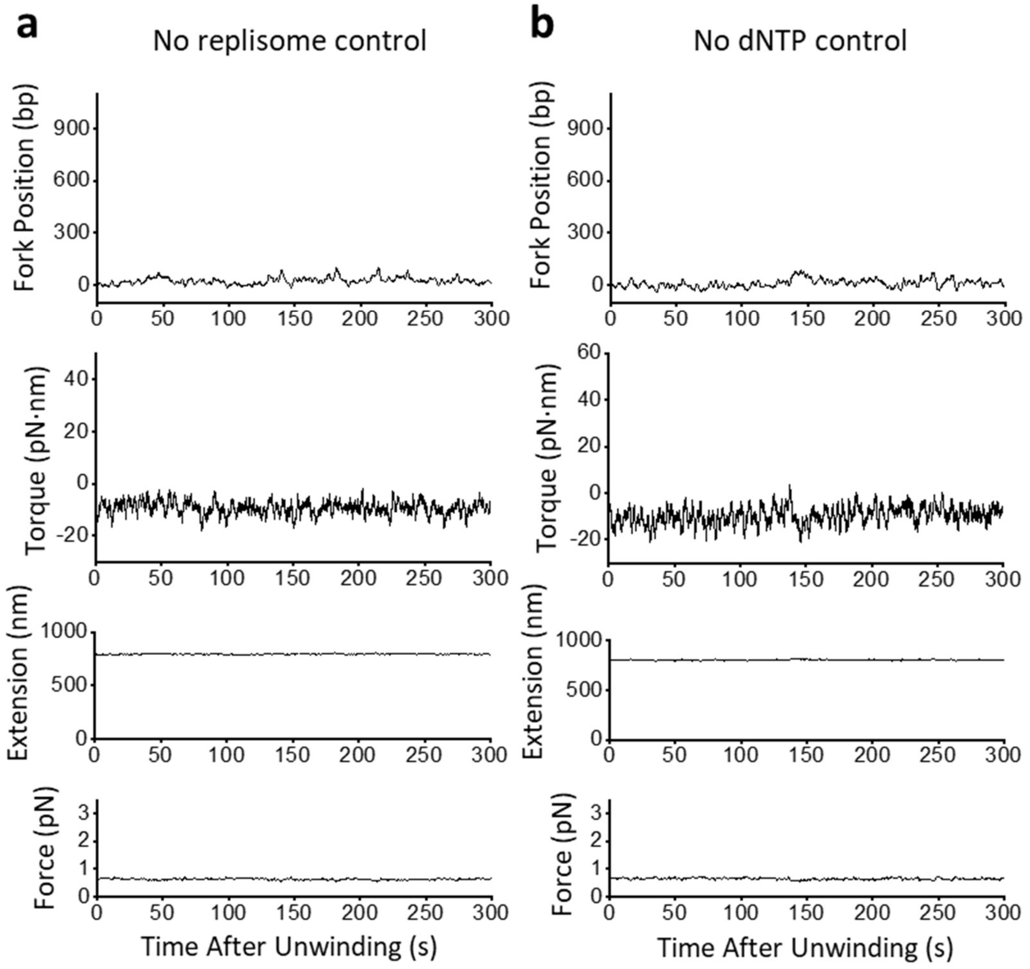

Supplementary Fig. 6. Control experiments without the replisome or dNTP.

We performed two controls for the data taken in Fig. 2b. These experiments were carried out using the same experimental procedures as those of Fig. 2b, except by omitting either the replisome or dNTP. Since active replication requires both, neither of these conditions were expected to support any active fork progression. These data provide the baseline for those in Fig. 2b.

a. An example trace of the control without replisome. All signals remain nearly constant over time, with minimal fork motion. This observation is confirmed in a total of N = 16 traces.

b. An example trace of the control without dNTP. All signals remain nearly constant over time, with minimal fork motion. This observation is confirmed in a total of N = 15 traces.

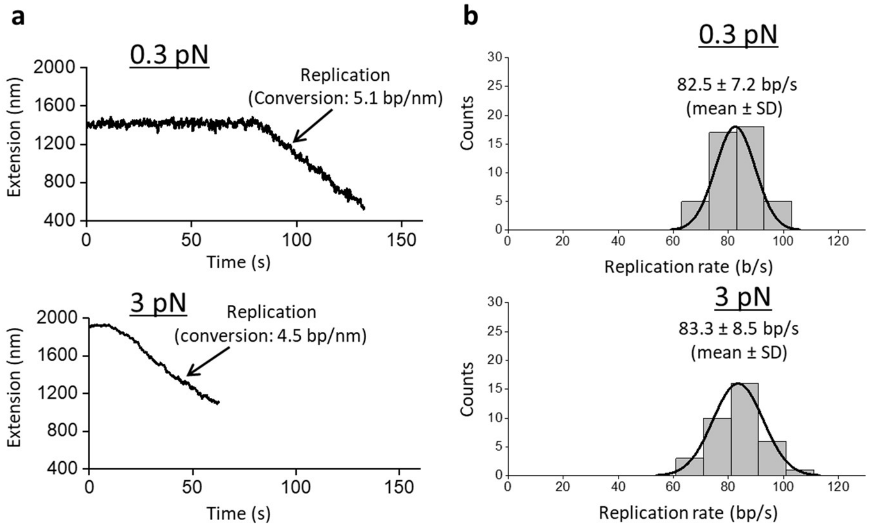

Supplementary Fig. 7. Force on the Y-shaped DNA substrate does not impact replication rate.

During a replisome stalling measurement using the AOT (Figs. 2 and 3), a force is exerted on the Y-shaped DNA substrate in addition to torque and may slow the replication speed. To investigate this possibility, we conducted the following experiments using the MT. In these experiments, we hold a Y-shaped DNA substrate under a specified force. After verifying a DNA tether was torsionally constrained, we introduced to the sample chamber nicking enzyme Nt.BspQI (NEB, Ipswich, MA) that has a target site on the parental DNA. This treatment torsionally un-constrains the tether, and subsequent replication cannot accumulate torsion. We then investigate how the exerted force impacted the replication rate by monitoring the DNA extension.

a. Example traces under 0.3 and 3 pN force. Replication decreases the DNA extension since the ssDNA of the lagging strand has a large stiffness[9,10]. A trace is terminated if the replication encounters a nicking site. The conversion factor from extension change to base pairs replicated is also indicated.

b. Histograms of replication rates under 0.3 and 3 pN force. The mean replication rates are in good agreement with each other, indicating that the applied force, ranging from 0.3 to 3 pN used in all our experiments, does not impact replication significantly.

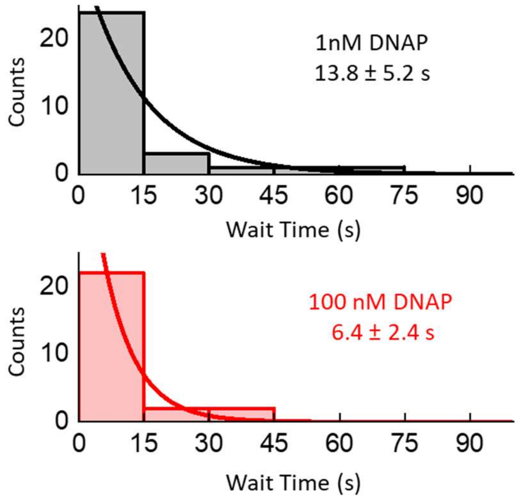

Supplementary Fig. 8. Wait-time histograms of 1 nM and 100 nM DNAP measured using the MT under 0.5 pN force. During an experiment, the Y-shaped DNA substrate is unwound using the MT (Methods). Initial DNAP replication activity is evidenced by an increase in DNA extension as DNAP removes the (-) supercoiling. Each histogram is fit by an exponential function. The mean wait time from the fit and the uncertainty in the fit parameter are shown. These histograms show that DNAP can locate the fork rapidly (~ 13 s) even at 1 nM DNAP concentration.

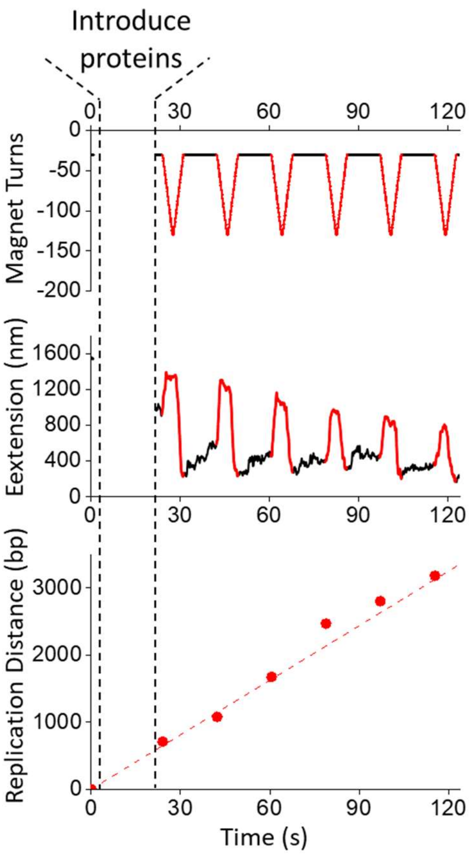

Supplementary Fig. 9. An example trace illustrating the method to track the replication fork position in the presence of 10 nM gyrase shown in Fig. 6. After the protein introduction, we obtained the extension-turns relation by rapidly unwinding and rewinding the DNA (red regions). The zero-torsion state is located at the maximum extension, which can then be used to determine the replication fork position (Methods). These checks were carried out every 18 seconds. As the replication proceeds, the parental DNA shortens, leading to a concurrent shortening of the extension with a conversion factor of 0.2372 nm extension decrease for each base pair replicated. A linear fit to the replication distance is also shown to guide the eye.

Supplementary References

1. Gao, X., Hong, Y., Ye, F., Inman, J. T. & Wang, M. D. Torsional Stiffness of Extended and Plectonemic DNA. *Phys. Rev. Lett.* **127**, 028101 (2021).
2. Deufel, C. & Wang, M. D. Detection of Forces and Displacements along the Axial Direction in an Optical Trap. *Biophys. J.* **90**, 657–667 (2006).
3. Le, T. T. *et al.* Synergistic Coordination of Chromatin Torsional Mechanics and Topoisomerase Activity. *Cell* **179**, 619-631.e15 (2019).
4. Lee, J. *et al.* Chromatinization modulates topoisomerase II processivity. *Nat. Commun.* **14**, 6844 (2023).
5. Forth, S. *et al.* Abrupt Buckling Transition Observed during the Plectoneme Formation of Individual DNA Molecules. *Phys. Rev. Lett.* **100**, 148301 (2008).
6. Forth, S., Sheinin, M. Y., Inman, J. & Wang, M. D. Torque measurement at the single-molecule level. *Annu. Rev. Biophys.* **42**, 583–604 (2013).
7. Ma, J., Bai, L. & Wang, M. D. Transcription Under Torsion. *Science* **340**, 1580–1583 (2013).
8. Ma, J. *et al.* Transcription factor regulation of RNA polymerase's torque generation capacity. *Proc. Natl. Acad. Sci.* **116**, 2583–2588 (2019).
9. Inman, J. T. *et al.* DNA Y structure: a versatile, multidimensional single molecule assay. *Nano Lett.* **14**, 6475–6480 (2014).
10. Killian, J. L., Inman, J. T. & Wang, M. D. High-Performance Image-Based Measurements of Biological Forces and Interactions in a Dual Optical Trap. *ACS Nano* **12**, 11963–11974 (2018).
