# Supplementary material for: Torsion is a dynamic regulator of DNA replication stalling and reactivation
Source: Nat Commun. 2025 Nov 26;16:10543. doi: 10.1038/s41467-025-65567-5 (PMC12658067; doi:10.1038/s41467-025-65567-5)
Supplement: Supplementary file 3 — Description of Additional Supplementary Files [file 41467_2025_65567_MOESM3_ESM.pdf]

Description of Additional Supplementary Files

Supplementary Movie 1. Real-time visualization of replisome rotation of DNA. This video animation shows an example trace of replisome rotation of DNA under 12.6 pN·nm torque as shown in Fig. 1d. The replisome rotation is visualized via the rotation of the trapped nanofabricated quartz cylinder in the AOT, as the cylinder rotates to follow the replisome rotation of the DNA. The inset circle represents the top view of the cylinder with its angular orientation indicated by the red arrow. Only a portion of this trace (from 40 s to 55 s) is animated. As shown, continuous replication (red regions of the curve) is interrupted by pauses (black regions of the curve). The scale bar provides the conversion from turns to the translocation distance of the replisome.
